# Innocent Body-Shadow Mimics Physical Body

**DOI:** 10.1177/2041669517706520

**Published:** 2017-05-01

**Authors:** Kenri Kodaka, Ayaka Kanazawa

**Affiliations:** Nagoya City University, Aichi, Japan

**Keywords:** body perception, multisensory or cross-modal processing, body-shadow, proprioception

## Abstract

The paradigm of the rubber hand illusion was applied to a shadow to determine whether the body-shadow is a good candidate for the alternative belonging to our body. Three kinds of shadows, a physical hand, a hand-shaped cloth, and a rectangle cloth, were tested for this purpose. The questionnaire results showed that both anatomical similarity and visuo-proprioception correlation were effective in enhancing illusory ownership of the shadow. According to the proprioceptive drift measurement, whether the shadow purely originated from the physical body was a critical factor in yielding the significantly positive drift. Thus, results demonstrated that the shadow can distort illusory ownership with the rubber hand illusion paradigm, but the proprioception was clearly distorted only when the body-shadow was purely applied. This implies the presence of special cognitive processing to discriminate the self-body shadow from the others.

## Introduction

There is a well-known and old game for children called “ka-ge-fu-mi” in Japan wherein a chaser (tagger) tries to step on the shadow of the others (resulting in switching of roles in the game). In this imaginary world, touching the body-shadow is identical to touching the physical body. Similar beliefs, such that an attack to the body-shadow is equivalent to physical body, have been found in various parts of the world, suggesting a special involvement with the body-shadows in cognitive processing.

Although seeing a shadow of an object is found to have a positive effect on recognizing the object accurately in many studies (e.g., Castiello, 2001; Elder, Trithart, Pintilie, & MacLean, 2004; Rensink & Cavanagh, 2004) or even to lead to a misunderstanding of the object’s trajectory (e.g., Kersten, Mamassian, & Knil, 1997), fewer studies have examined the effect of seeing the body-shadow. Exceptionally, Pavani and colleagues have been continuously exploring the characteristics of the body-shadow in a visuo-tactile interference situation for more than a decade (Pavani & Castiello, 2004; Pavani & Galfano, 2007; Pavani, Rigo, & Galfano, 2014). In their research, merely seeing a single hand-shadow was found to cue tactile attention to the physical hand casting the shadow, compared with the other hand; importantly, this cueing effect significantly declined when the shadow was transformed into a polygonal shape or when it was replaced with a static hand-shaped hand picture. In addition, the polygonal shadow was also found to develop a cueing effect over time, probably due to the spatio-temporal correlation between the hand and the shadow. These results, as has been clearly discussed in their respective studies, remind us of the relation between a tool and physical hand when using the tool in an active way; the tool use extends the visual receptive field to represent the peri-personal space so that the tool is encoded as an extension of the body (Iriki, Tanaka, & Iwamura, 1996; Witt, Proffitt, & Epstein, 2005; Yamamoto & Kitazawa, 2001). A recent report by Kuylen, Balas, and Thomas (2014), showing that subjects underestimate the distance to a target when the body-shadow is cast in the direction of the target on the ground, is a further direct demonstration of the validity of this analogy. In this way, recent limited studies on the body-shadow implied that the cognitive processing of the body-shadow is overlapped with that of the physical body in the brain.

Therefore, it is probable that the body-shadow is processed as a substitution or extension of the physical body casting that shadow, but it is so difficult to assume that the participants in the related experiments felt their shadow just as a part of the body amid the relevant perceptual distortion. In other words, the related studies have demonstrated that the body-shadow can affect the cognitive processing in a level of body-schema but not in a level of body image (Gallagher, 2005). This seems obvious, considering that we do not misunderstand the body-shadow as a part of the body in general situations. The question then is whether there is a way to somehow translate the body-shadow into an alternative belonging to our physical body?

An obvious feeling we naturally have for each part of the self-body is called body ownership. Rubber hand illusion (RHI) can provide body ownership to a fake hand based on spatio-temporal correlation between visual and tactile stimulation (Botvinick & Cohen, 1998). Another type of RHI that has attracted increasing interest over the last few years is where we see a rubber hand on the top of a desk moving in sync with the movement of a hand under a desk, called moving RHI. Researchers have somehow controlled an artificial hand (hand model, computer graphics, and a robot hand) coordinated with the hand movement, thus completing the visuo-proprioception correlation (e.g., Braun, Thorne, Hildebrandt & Debener, 2014; Caspar, Cleeremans, & Haggard, 2015; Kalckert & Ehrsson, 2012; Slater, Perez-Marcos, Ehrsson, & Sanchez-Vives, 2009). Naively speaking, the body-shadow seems a quick way to realize such a correlation in a more natural manner considering that the motion of the body-shadow is originally associated with the physical body; we do not need to add anything for the visuo-proprioception correlation. In addition, RHI is found to be applicable even to two-dimensional hand appearance (e.g., captured video in Pavani & Zampini, 2007; hand drawing in Pasqualotto & Proulx, 2015). Despite the body-shadow satisfying the requirements for the ownership distortion, no research has been directly focused on the body-shadow in the RHI paradigm.

**Figure 1.**
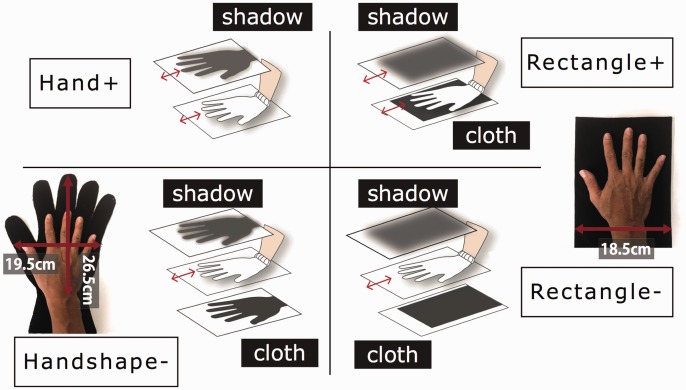
Four kinds of shadows were tested in the experiment where only the active (+) shadow on the top screen moved in sync with the hand movement on the middle plate. In the static (−) shadow, the hand-shaped or rectangle cloth, which was large enough to cover the physical hand movement on the middle plate, was placed on the bottom plate.

In this research, four shadow environments were designed for illusion induction to examine the effect of body-shadow in RHI (Figure 1). Specifically, Hand+ and Handshape− showed the hand’s or hand-shaped cloth’s shadow, whereas Rectangle+ and Rectangle− showed the shadow of rectangle cloth. In addition, the shadow of Hand+ and Rectangle+ (active shadows) moved in sync with the hand movement, whereas Handshape− and Rectangle− (static shadows) remained stable regardless of the hand movement. The participant was asked either to move the physical hand back and forth (active trial) or to not move (static trial) in the illusion induction. The strength of the body ownership toward the shadow was analyzed on the basis of the questionnaire rating and the proprioceptive drift measurement.

## Results

First, ownership statements for both static and active trials were analyzed using a three-way ANOVA with within-subject factors: *shadow type* (Hand+, Handshape−, Rectangle+, Rectangle−), *hand movements* (static trial vs. active trial), and *statement type* (illusion vs. control). The results (see top of Figure 3) revealed a significant main effect of *shadow type*, *F*(3,45) = 20.40, *p* < .001, and *statement type*, *F*(1,15) = 51.84, *p* < .001, in addition to a significant interaction among the three factors, *F*(3,45) = 14.03, *p* < .001. *Hand movement* did not yield any significant effect, *F*(1,15) = 0.00, *p* = .98, n.s. A follow-up test with simple effects analysis revealed that the static trial was accompanied by a significant predominance of the average rating for the ownership illusion statements over that of the ownership control statements for the three shadows, Hand + : *F*(1,120) = 52.65, *p* < .001; Handshape−: *F*(1,120) = 21.41, *p* < .001; Rectangle + : *F*(1,120) = 4.88, *p* < .03; Rectangle − : *F*(1,120) =2.83, *p* = .10, n.s., whereas such a significant trend was only shown in the active shadows in the active trial, Hand+: *F*(1,120) = 104.05, *p* < .001; Handshape−: *F*(1,120) = 0.62, *p* = .43, n.s.; Rectangle+: *F*(1,120) = 35.93, *p* < .001; Rectangle−: *F*(1,120) = 0.04, *p* = .84, n.s. In addition, multiple comparisons using Ryan’s method (Horn, 1981) revealed that the hand shadow had significantly greater scores for the illusion ownership rating than the rectangle shadow in the static trial (*p* < .001 for Hand+ vs. Rectangle+ and Handshape− vs. Rectangle−). Finally, the active trial provided significantly higher ratings for the illusion ownership statements as compared with the static trial for the active shadows, *F*(1,120) = 11.80, *p* < .001 for Hand+; *F*(1,120) = 27.55, *p* < .001 for Rectangle+), whereas a significant trend toward the opposite direction was found for the static shadows, *F*(1,120) = 28.24, *p* < .001 for Handshape−; *F*(1,120) = 7.41, *p* < .008 for Rectangle−.

Next, agency statements for the active trial were analyzed using a two-way ANOVA with within-subject factors *shadow type* and *statement type* and revealed a significant main effect of *shadow type*, *F*(3,45) = 20.4, *p* < .001, and *statement type*, *F*(1,15) = 51.84, *p* < .001, in addition to significant interaction, *F*(3,45) = 14.37, *p* < .001. Simple effects analysis revealed that the active trial was accompanied by a significant predominance of the average rating for the agency statements over that of the agency control statements only for active shadows, *F*(1,60) = 19.58, *p* < 0.001 for Hand + ; *F*(1,60) = 28.87, *p* < .001 for Rectangle+.

Finally, we analyzed proprioception change. Prior to the analysis, the proprioceptive drift of each participant was calculated for each sight condition (eyes-open and eyes-closed) using a common pre-illusion proprioception of the right hand (baseline), which was pooled and averaged per sight condition to restrain dispersion. The obtained proprioceptive drifts (post-illusion proprioceptive height minus pre-illusion proprioceptive height) were analyzed using a three-way ANOVA with within-subject factors *shadow type*, *hand movement*, and *sight type* (eyes-open vs. eyes-closed). The results (see lower left of Figure 3) revealed a significant main effect of *shadow type*, *F*(3,45) = 5.56, *p* < .003, and *sight type*, *F*(1,15) = 6.95, *p* < .019, whereas *hand movement* did not yield a significant effect, *F*(1,15) = 0.57, *p* = .46, n.s. In addition, the multiple comparisons (Ryan’s method) under the eyes-open measurement demonstrated that the drift for Hand+ was significantly higher than that of the three other shadows (*p* < .004 vs. Handshape − , *p* < .001 vs. Rectangle+, and *p* < .001 vs. Rectangle−). Finally, one-tailed Welch’s *t*-test comparison with zeroes showed that Hand+ had a significant positive drift in both the static and active trials with the eyes-open measurement but not with the eyes-closed measurement, *t*(15) = 4.01, *p* < .002 for static trial; *t*(15) = 3.55, *p* < .003 for active trial, whereas the other shadows did not have any significant effect.

**Figure 2.**
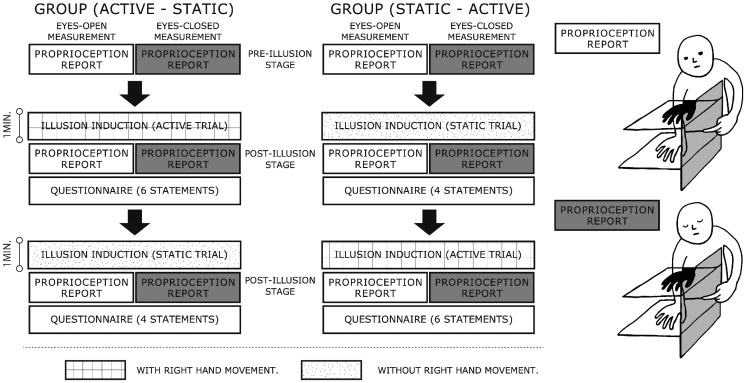
This diagram shows the experimental procedure for one session. The static and active trials were held one after the other following the common pre-illusion proprioception measurement. Each static or active illusion induction follows the post-illusion proprioception measurement and the questionnaire. The order of the trials in a session was counterbalanced among participants.

**Figure 3.**
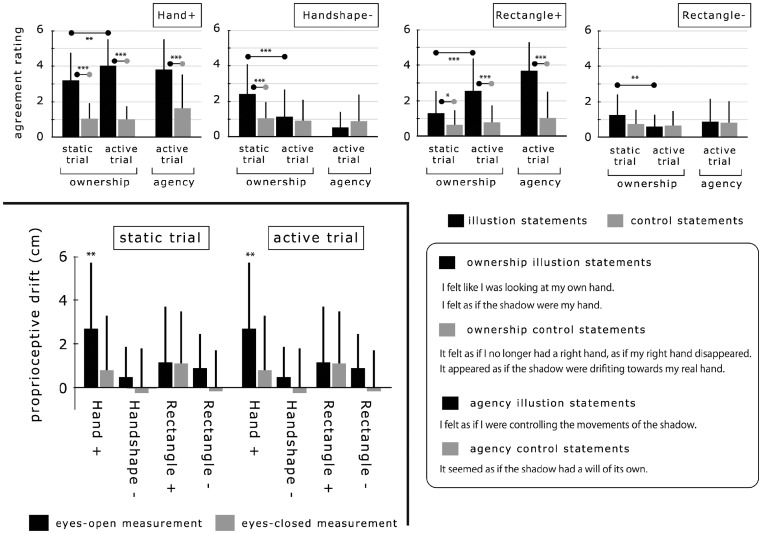
The top graphs represent the results of the questionnaire agreement, where all statistical significances were calculated based on ANOVA. The bottom graphs represent the results of the proprioceptive drift where only the Hand+ with gaze measurement yielded a significant positive drift compared with zero, using the paired two-tailed *t*-test (*p < .05, **p < .01, ***p < .001).

## Discussion

The questionnaire analysis revealed that both the anatomic similarity to the body-part and the visuo-proprioception correlation were effective in enhancing illusory ownership. This demonstrates that the shadow could distort illusory ownership with the RHI paradigm. In contrast, these effects on the proprioceptive drift were uncertain. In fact, there was no significant difference in the drift between the static and active trials, regardless of the type of the shadow shape. In addition, the hand-shaped cloth’s shadow (Handshape−) did not yield a significantly greater drift than the rectangle cloth’s shadows in any situations. Thus, the proprioceptive drift was less sensitive to both the anatomic similarity and the visuo-proprioception correlation as compared with the illusory ownership. These points support the recent view that the proprioceptive distortion does not have a causal link with a feeling of limb ownership (Abdulkarim & Ehrsson, 2016).

As is unusual for the study of RHI, we tested the proprioceptive drift measurement not only in the eyes-closed situation but also with eyes open toward the shadow (eyes-open measurement) to probe the effect of seeing the shadow in detail. Contrary to the former reports with the other artificial hands, all the shadows, including the body-shadow, did not yield the significant positive drift in the eyes-closed measurement, even for the active trial. Although we do not have a convincing explanation for this disagreement, it is invalid to suppose that this is consistently reproducible considering that we found the opposite trend for the body-shadow in some preliminary experiments. With the eyes-open measurement, the physical hand’s shadow (Hand+) was the only shadow that provided with the significant positive drift; notably, its drift was also significantly greater than for the hand-shaped shadow. Thus, the proprioceptive drift did not occur merely because the subjects see the hand-shaped shadow; the shadow should be the innocent body-shadow. Here, it is also remarkable that even the static trial yielded the significant positive drift toward the body-shadow. As the static trial did not involve the physical hand movement, the results indicate that just seeing the body-shadow results in visuo-proprioceptive recalibration. In the study of RHI, a few studies have reported that changes in hand proprioception can occur even without visuo-tactile correlation (e.g., Durgin, Evans, Dunphy, Klostermann & Simmons, 2007; Rohde, Di Luca, & Ernst, 2011). Considering that merely hand-shaped shadow did not yield such a distortion, it is valid to suppose that there is a special screening to join the group of artificial hand capable of inducing the drift just by seeing; the body-shadow seemingly has this membership. In summary, the critical factor to yield the significant distortion of the proprioception was determined by whether or not the shadow had purely originated from the physical body, implying the presence of special cognitive processing to discriminate the self-body shadow from the others.

## Methods

In total, 16 students (four females and 12 males, 18–27 years) participated in the experiment. All participants were recruited from the Faculty of Design and Architecture, Nagoya City University, and signed informed consent prior to their participation. All participants received a book of tokens as compensation (1000 Yen). All experiments were conducted in accordance with the Declaration of Helsinki. The study protocols were approved by the ethics committee of the Nagoya City University.

We set up three acrylic transparent plates vertically at a suitable interval, where the participant could see a shadow from any of the three kinds of objects (a right hand, a hand-shaped cloth, or a rectangle cloth). They could be viewed on the screen of the top plate via a light source installed on the floor, with the palm of the right hand on the middle plate, 12 cm below the top plate. The shadow on the top screen approximately magnified the objects below by 5% to 10%. Four shadow environments were designed for illusion induction: first, an active hand shadow (Hand+) where the top plate shows the right hand’s shadow in a straightforward manner; second, a static, hand-shaped shadow (Handshape−) showing the shadow of the hand-shaped cloth (on the bottom panel) that remained stable regardless of the hand movement; third, an active rectangle shadow (Rectangle+) showing the shadow of a rectangle cloth (clinging to the right hand) that moved in sync with the hand movement; and fourth, a static rectangle shadow (Rectangle−) showing the shadow of the rectangle cloth (on the bottom panel) that remained stable regardless of the hand movement (Figure 1).

Figure 2 depicts the experimental procedure for one session. At the beginning of each session, the experimenter positioned the right hand of the blindfolded participant in place on the middle plate. Next, the participants were asked to indicate at what height they felt their right hand was by touching a board attached to the left side of the acrylic plates. To identify the position of each participant’s left index finger, the board was covered with a sheet of paper with a millimeter grid and the experimenter used a pen to mark the position corresponding to the central part of the participant’s fingernail on this paper. The measurements were classified into two—eyes-closed and eyes-open, which were counterbalanced within each participant. Specifically, this proprioception report was done with the participants’ eyes closed (eyes-closed measurement) or with the eyes gazing toward the black cloth covering the top screen (eyes-open measurement). Next, they were asked to look at the shadow on the top screen for 1 min while not moving their hand (static trial) or moving their hand back and forth (active trial). Note that this factor—*hand movement* (static or active trial)—is independent from the classification of static or active shadows. Thus, the illusion induction was accompanied by the shadow’s movement only when the active trial was applied for the active shadows (Hand+ and Rectangle+); the static trial did not involve the shadow’s movement for not only the static shadows but also the active shadows. Subsequent to this illusion induction, they were asked again to localize the right hand with the sight condition that was applied at the pre-illusion stage. Notably, the eyes-open measurement was done without the participants taking their eyes off the shadow to measure proprioceptive height during the prior illusion induction as accurately as possible. Quickly after they answered four- or six-item questions (see lower right of Figure 3), including statements relating to ownership (for both trials) and agency (only for the active trial), the trial was switched to another one. Half of the participants first took the static trial and second the active trial during a single session, whereas the remaining participants took the opposite. All participants experienced eight sessions (two sight conditions × four shadow conditions), which were allocated randomly to the participants but with a constraint that the same sight or shadow condition was not applied twice in a row. At least a 30-s break was taken at the end of each session to restrain the effect of the preceding condition.
